# NURSING HOME CONTEXTUAL FACTORS IMPACTING PALLIATIVE CARE CONSULTATION IMPLEMENTATION

**DOI:** 10.1093/geroni/igae098.4034

**Published:** 2024-12-31

**Authors:** Joan Carpenter, Elisha Oduro, Ruth Lopez, Kathleen Unroe

**Affiliations:** University of Maryland Baltimore, Baltimore, Maryland, United States; University of Maryland Baltimore, Baltimore, Maryland, United States; MGH Institute of Health Professions, Boston, Massachusetts, United States; Indiana University, Indianapolis, Indiana, United States

## Abstract

Palliative care (PC) consultation has been shown to improve the quality of life of nursing home (NH) residents. However, its implementation in NHs is uneven and poorly characterized. Grounded in the Integrated Promoting Action on Research Implementation in Health Services framework, we used a qualitative descriptive approach to explore how the inner context (organization leadership, staff, culture) and the outer context (policy, finances) impact the implementation of PC consultation in NHs. We conducted semi-structured interviews with key partners in fourteen NHs in the Eastern US. We used directed content analysis to analyze transcripts and identify key themes. Participants (N=33) were NH leaders, nurses, social workers, and palliative care agency leaders and nurse practitioners, predominantly female (93.9%), White (93.9%), and one-third had at least 20 years of experience working in NHs. Our findings reveal factors within the inner and outer contexts that either pull consultations toward or push consultations away from residents. In the inner context, factors that pull consultations toward residents were supportive organizational leadership, staff referrals with detailed residents’ information and assistance during consultations, and a welcoming NH environment. However, the absence of a reliable screening tool for staff to identify residents for consultation was seen as pushing away from residents. In the outer context, policies that levy monetary penalties for hospital readmission were seen to pull consultations toward residents while financial “burdens” and “drains” were seen as pushing consultations away from residents. These fndings will be used to develop a sustainable and scalable PC consultation model in NHs.

